# Unveiling immunity gaps and determining a suitable age for a third dose of the measles-containing vaccine: a strategic approach to accelerating measles elimination

**DOI:** 10.1016/j.lansea.2024.100523

**Published:** 2024-12-21

**Authors:** Somya Mehra, Sajikapon Kludkleeb, Chutikarn Chaimayo, Pornsawan Leaungwutiwong, Saranath Lawpoolsri, Wirichada Pan-ngum, Kulkanya Chokephaibulkit, Thundon Ngamprasertchai

**Affiliations:** a Mahidol Oxford Tropical Medicine Research Unit, Mahidol University, Bangkok, Thailand; b Department of Clinical Tropical Medicine, Faculty of Tropical Medicine, Mahidol University, Bangkok, Thailand; c Department of Microbiology, Faculty of Medicine Siriraj Hospital, Mahidol University, Bangkok, Thailand; d Department of Microbiology and Immunology, Faculty of Tropical Medicine, Mahidol University, Bangkok, Thailand; e Department of Tropical Hygiene, Faculty of Tropical Medicine, Mahidol University, Bangkok, Thailand; f Department of Pediatrics, Faculty of Medicine Siriraj Hospital, Mahidol University, Bangkok, Thailand; g Siriraj Institute of Clinical Research, Faculty of Medicine Siriraj Hospital, Mahidol University, Bangkok, Thailand

**Keywords:** Measles, Immunity gaps, Measles vaccine, Third dose measles vaccine, Seropositivity

## Abstract

**Background:**

In highly measles immunized countries, immunity gaps in adolescents and young adults are a key issue posing an obstacle to measles elimination. This study aims to identify the gaps by estimating the age-stratified probability of seropositivity, and to ascertain a suitable age for the administration of a third dose of a measles-containing vaccine (MCV3) to effectively fill these gaps.

**Methods:**

We retrospectively obtained measles serological results from hospital setting among among individuals aged 13–39 years and developed a serocatalytic dynamic probability model, stratifying seropositivity due to vaccination or natural infection. We calibrated the model to age-stratified seropositivity data within a Bayesian setting using the Metropolis–Hastings algorithm. A scenario analysis to determine a suitable age for MCV3 administration was also performed.

**Findings:**

The overall prevalence of measles seropositivity was 65.6% (95% confidence interval [CI]: 61.5–69.6). Posterior predictive curves for the age-stratified seroprevalence exhibited a decreasing trend from ages 13–20 years but an upward trend from 26 to 30 years. The age at which a given individual’s serostatus reached a 50% probability of seronegativity was found to be approximately 18–20 years depending on the annual measles force of infection.

**Interpretation:**

Our findings highlight a significant measles immunity gap in young adults aged 20–26 years, posing an increased risk of transmission. A MCV3 at the age of 18–20 years potentially closes the gap and aids measles elimination programmes.

**Funding:**

This work was supported by Faculty of Tropical Medicine (MCTM, ICTM grant), 10.13039/501100004156Mahidol University (to T.N.) and APC fee was supported by 10.13039/501100004156Mahidol University (to T.N.). S.M. and W.P. were funded in whole, or in part, by the 10.13039/100010269Wellcome Trust [Grant number 220211]. For the purpose of open access, the authors have applied a CC BY public copyright licence to any Author Accepted Manuscript version arising from this submission.

**Research in context**

**Evidence before this study**

The Immunization Agenda 2030 underlines the importance of effective measles surveillance to detect immunity gaps and prevent outbreaks, aiding in the effort to eradicate the disease globally. Supplemental immunization efforts, including catch-up vaccination campaigns, are critical for increasing coverage. Despite high vaccination rates in some regions, immunity gaps persist as a significant issue. Additionally, the introduction of a third measles vaccine dose (MCV3) may be an actionable approach in areas with restricted surveillance capacities, particularly for individuals in high-risk areas.

**Added value of this study**

A significant measles immunity gap was identified in young adults aged 20–26 years, increasing transmission risk. Administering a third dose of measles vaccine at 18–20 years of age could potentially close this gap and enhances elimination programs.

**Implications of all the available evidence**

Administering a third dose of the measles vaccine (MCV3) to those aged 18–20 could strengthen efforts to eliminate measles. Countries may customize their approach to close immunity gaps by routinely offering MCV3 to everyone or focusing on high-risk groups.

## Introduction

Achieving high population immunity is crucial for measles transmission elimination. However, global elimination strategies were disrupted during the COVID-19 pandemic, resulting in more than 22 million infants missing their first measles vaccine dose—the largest increase in two decades.^1^ The estimated global coverage for the first vaccine dose declined to 81%,^2^ and measles cases and associated fatalities rose by 68% during 2021–2022. This increase can be attributed to reduced vaccine coverage, the cessation of COVID-19 preventive measures, and inadequate surveillance systems.^2^^,^^3^ Although high vaccine coverage is reported in some countries, immunity gaps can still arise, leading to suboptimal protection. These gaps may be caused by waning vaccine-induced immunity^4^ or low vaccine uptake, posing a subsequent risk of outbreaks.^5^ Factors such as the age at first MCV dose and whether the setting is endemic or elimination may influence the duration and rate of waning immunity.^4^^,^^6^ In Thailand, measles surveillance from 2021 to 2023 reported 64 to 66 confirmed measles cases annually. As of 2023, the national immunization schedule includes the Measles, Mumps, and Rubella (MMR) vaccine administered at 9 months and 1.5 years of age. Additionally, the Measles and Rubella (MR) vaccine is provided to healthcare workers and first-year students in medical and public health faculties. The coverage rates for the first dose (MCV1) and second dose (MCV2) of a measles-containing vaccine were 93% and 87%, respectively, in 2023.^7^

The Immunization Agenda 2030 emphasizes the necessity of robust measles surveillance systems for identifying immunity gaps,^8^ which is essential for preventing outbreaks and progressing towards global elimination. Effective surveillance systems, along with achieving a minimum of 95% coverage for two doses of the measles-containing vaccine (MCV), are critical strategic approaches.^3^ However, these efforts face challenges, such as disruption to routine immunization services due to pandemics or political instability. To mitigate these challenges, supplemental immunization activities (SIAs), such as catch-up vaccination campaigns, play a vital role in bridging coverage gaps.^9^ Furthermore, implementing a third dose of MCV (MCV3) at a specific age–especially for individuals or those residing in high-risk regions–may be a feasible strategy in settings where surveillance capabilities are limited.

Current guidelines advocate for the administration of MCV3 to individuals who have previously received two doses but are at increased risk during outbreaks of measles or mumps.^7^^,^^10^^,^^11^ This encompasses military service members, university students, international travelers and women of childbearing age, particularly in the absence of verifiable immunization records.^10^^,^^11^ Administering a third measles-containing vaccine (MCV3) dose to individuals who have completed the standard two-dose regimen can enhance immunogenicity, particularly in those with waning immunity. Studies show that MCV3 boosts both humoral and cellular responses, increasing measles-specific antibodies and promoting long-lasting immunity. This third dose may also provide extended protection, potentially reducing the need for future boosters. Additionally, MCV3 could be valuable in outbreak settings or among high-risk populations, supporting its use as a targeted strategy for maintaining effective seroprotection alongside its established safety and tolerability profile in young adults.^12^, ^13^, ^14^, ^15^ The administration of MCV3 at a suitable age is essential for closing immunity gaps and mitigating the risk of outbreaks. This could serve as an effective strategy for countries with inadequate surveillance systems or low coverage of MCV2, particularly when the affordability and favorable safety profile of the vaccine are considered. This study aims to identify immunity gaps by estimating the age-stratified probability of seropositivity, and to determine a suitable age for MCV3 administration to effectively close the gaps using a model-based approach.

## Methods

### Study population and sample sizes

This multi-centre cross-sectional study was carried out in university-based hospitals between July 2022 and December 2023 and aimed to extract measles serological data from the years 2018–2021. Participants included Thai young adults, aged 13 to 39, who were visiting for screening for measles serology primarily undergoing measles serology screening due to requirements for healthcare personnel or international study programs. Seropositivity was defined as an optical density (OD) ratio greater than 1.1 as determined by the enzyme-linked immunosorbent assay (ELISA) technique, while a ratio of 1.1 or less was considered negative.^16^ Baseline demographic data and records of measles-containing vaccines (MCV) administration were collected. Sample sizes were determined using measles seropositivity reported in the serological surveillance data from 2004 to 2014. It was estimated to range between 73% and 99%^17^^,^^18^ across each decade age group for individuals aged 15 and older in Thailand. Consequently, a sample size of 138 was necessary for each 10-year age group. In total, a minimum of 400 serological samples were collected from participants aged 13 to 39.

### Model structure

**Fig. 1:**
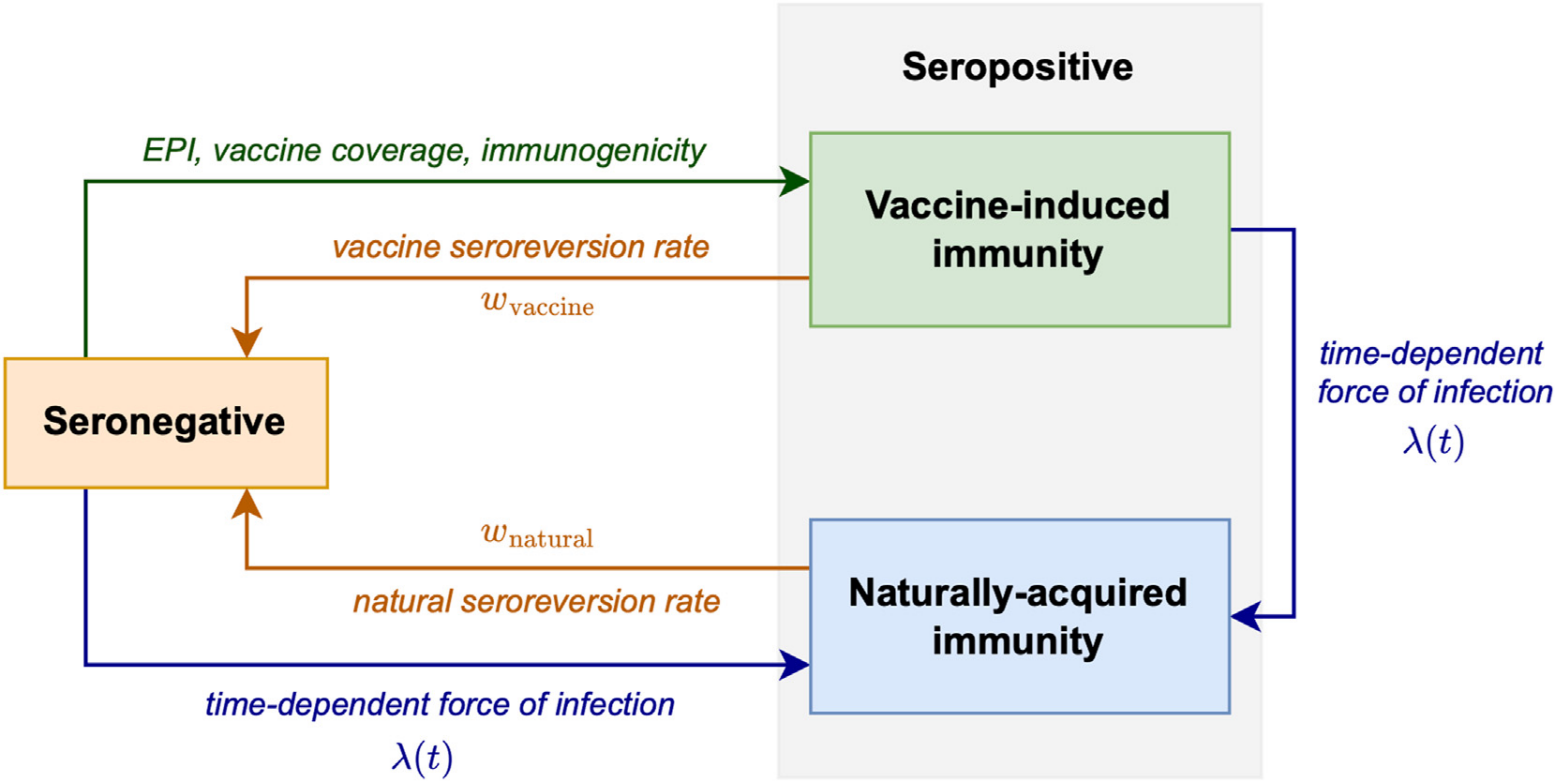
Schematic diagram of the serocatalytic model.

A serocatalytic dynamic probability model was developed, stratifying seropositivity by age due to either vaccination or natural exposure for individual subjects. Our study defined natural exposure or natural immunity as immunity resulting from prior infection. A schematic of the model is shown in Fig. 1. Maternal immunity was excluded from consideration, given that the initial age of interest was 13 years.^19^ As such, each individual was assumed to be seronegative at birth. Seroconversion could occur due to either vaccination, or natural exposure. We modelled different rates of seroreversion for individuals who acquired seropositivity through natural exposure $\left(w_{\text{natural}}\right)$, as opposed to vaccination only $\left(w_{\text{vaccine}}\right)$.^20^ Natural immunity was expected to confer a longer average duration of protection $1/w_{\text{natural}}$ than vaccine-induced immunity $1/w_{\text{vaccine}}$.^21^

Vaccination was modelled in accordance with the Thailand Expanded Program Immunization (EPI) described in the Supplementary Material. For an individual born in year *b*, scheduled to receive dose *i* of a MCV at age $A_{i}$ under the EPI, we first computed the probability of vaccine-induced seroconversion $c_{i}^{\left(b\right)}$ associated with dose *i*. This was taken to be the product of MCV effectiveness (assumed to be 90% per dose for the measle vaccine, and 95% per dose for the Measles-Mumps-Rubella (MMR) vaccine^6^^,^^22^), and vaccine coverage in the year $\left(b+A_{i}\right)$, as reported by the Thai government.^6^^,^^23^ In case of missing coverage data, values were imputed using the average of the preceding and following years. Between the ages of $A_{i}$ and ($A_{i+T)}$, vaccine-induced seroconversion was then modelled to occur at the constant rate

$$r_{i}^{\left(b\right)}=-\frac{\log\left(1-c_{i}^{\left(b\right)}\right)}{T}\text{,}$$

Back-calculated to yield a cumulative seroconversion probability of $c_{i}^{\left(b\right)}$ (vaccine induced seroconversion at rate $r_{i}^{\left(b\right)}$ for a period of time T yields a seroconversion probability of $1-e^{{-r}_{i}^{\left(b\right)}T}=c_{i}^{\left(b\right)}$, as desired); this is a standard construction to yield smooth model-derived probabilities of seropositivity.

Rather than modelling disease transmission directly, we assumed a functional form for the rate of natural exposure to measles, referred to hereafter as the force of infection (FOI). Only a non-immune (seronegative) individual would develop the disease following exposure; a seropositive individual would instead receive an immunity boost. Prior to the initiation of the EPI in 1982,^7^ we assumed a constant FOI $\lambda_{\max}$. To allow for systematic declines in transmission resulting from the nationwide immunization program, the FOI was modelled to decay exponentially over time (measured in years AD) at rate α thereafter, yielding

$$\lambda \left(t\right)=\left\{ \begin{array}{l} \lambda_{\max}\;,t<1982\\ \lambda_{\max}\;e^{-\alpha \left(t-1982\right)},t\geq 1982 \end{array}\right.$$

Both α and $\lambda_{\max}$ are parameters to be estimated. The FOI governs the rate at which an individual who is either seronegative, or seropositive due to vaccine-induced immunity only, acquires natural immunity.

This approach neither accounted for age-dependence in the FOI, nor the elevated risk of exposure among specific populations, such as college or university students living in areas of high population density or household crowding.^24^ Governing equations are available in the Supplementary Material.

### Parameter estimation

We constructed a likelihood function adjusted for variation in sample sizes across age groups. The serostate of each individual was modelled as an independent Bernoulli random variable, with the probability of seropositivity dependent on both the year of birth and sample collection respectively. The Metropolis–Hastings algorithm was used to estimate parameters in a Bayesian setting (see Section 2 of the Technical Appendix for details). We took an informative Gamma prior (scale 1/3, shape parameter 3) for the maximal FOI $\lambda_{\max}$ for identifiability, and improper flat priors over the non-negative real line for all other parameters. We visually inspected trace plots and computed the Gelman-Rubin diagnostic $\hat{R}$ across 4 chains to assess convergence, ensuring $\hat{R}<1.02$ for each estimated parameter. Posterior predictive curves of age-stratified seroprevalence were generated by simulating serostates under 8000 posterior parameter combinations (sampled uniformly at random without replacement). Further details are provided in the Supplementary Material.

### Scenario analysis

A scenario-based approach was employed to ascertain a suitable age range for administering a MCV booster. This analysis was informed by the rates of seroreversion for naturally-acquired versus vaccine-induced immunity that were estimated by fitting the serocatalytic model to the seropositivity data. We examined measles seropositivity under the assumption that an individual experiences a constant FOI throughout their lifetime, using a spectrum of biologically plausible values. We modelled the probability of seropositivity as a function of age, assuming the administration of the first vaccine dose at one year and the second dose at six years, with a seroconversion probability of 95% for each dose. The analysis to determine the suitable age for booster MCV was based on a maximum threshold of seronegativity: following the second MCV dose at age six, we computed the minimum age at which the model-derived probability of seronegativity reached a pre-defined threshold. Reported age ranges for the MCV booster reflect uncertainty in our estimates for seroreversion rates. We additionally generated age-stratified seropositivity curves, accounting for the administration of a booster dose in the suggested age range, assuming that the rate of seroreversion for vaccine-induced immunity is equivalent for MCV1, MCV2, and MCV3.

### Ethics approval statement

The study was conducted according to good clinical practice of the Declaration of Helsinki and was approved by the Ethics Committee of Faculty of Tropical Medicine and Siriraj Hospital, Mahidol University (Protocol Number COA: MUTM 2022–040–01, MUTM 2022–040–02).

### Role of the funding source

The funders had no role in study design, data collection, data analysis, interpretation, writing of the report.

## Results

### Demographic characteristics

Table 1 presents the demographic characteristics and measles-specific IgG seropositivity of study population. The overall prevalence of measles seropositivity was 65.6% (95% confidence interval [CI]: 61.5–69.6). Young adults (aged 21–30 years) displayed the lowest seropositivity at 54.3% (95% CI: 47.6–61.1) while individuals aged 31–39 years demonstrated the highest seropositivity at 87.3% (95% CI: 82.3–92.4). A bar plot of sample counts and seropositivity by age can be found in the Fig. S4 of Supplementary Material.

**Table 1:** Demographic characteristics and measles-specific IgG seropositivity of study population (N = 543). Confidence intervals are based on bootstrap resampling (8000 iterations).

Characteristics	N (%)	Positive Measles-specific IgG %, (95% CI)
Age (years) at the time of blood collection		
13–20	177 (32.6)	59.3 (52.0, 66.7)
21–30	208 (38.3)	54.3 (47.6, 61.1)
31–39	158 (29.1)	87.3 (82.3, 92.4)
Mean age (SD) 25.3 (6.8)		Overall, 65.6 (61.5, 69.6)
Sex		
Male	175 (32.2)	68.6 (61.7, 75.4)
Female	368 (67.8)	64.1 (59.2, 69.0)
Occupation		
Healthcare personnel	232 (42.7)	50.4 (44.0, 56.9)
Non-healthcare personnel	118 (21.7)	59.3 (50.8, 67.8)
No data	193 (35.5)	N/A
Purpose of visiting		
Immunity screening prior to employment	269 (49.5)	49.1 (43.1, 55.0)
Presumed measles infection	78 (14.4)	66.7 (56.4, 76.9)
Others	196 (36.1)	87.8 (83.2, 92.3)

### Model fitting and parameter estimation

The expected duration of vaccine-induced immunity against measles was estimated to be 15.3 years (95% credible interval [CrI] 10.8–20.2); this can be formulated as a half-life, corresponding to a 50% probability of losing vaccine-induced immunity approximately every 10.6 years on average. In contrast, naturally-acquired immunity was estimated to be lifelong, with an average duration of 208 years (95% [CrI]; 119–418). Prior to the implementation of the immunization program in 1982, the force of infection (FOI) was estimated at 1.8 exposures per person per year on average (95% [CrI]; 0.75–3.73). Following the implementation, the FOI was modelled to decay exponentially, with an estimated rate of 0.21 per year (95% [CrI]; 0.15–0.28). Estimates for the FOI before 1982, and its rate of decay thereafter, were strongly correlated (see Supplementary Material): under the model and the estimated rate of seroreversion, similar seropositivity profiles could be attained under either a high initial FOI that decayed rapidly, or a lower initial FOI that decayed relatively slowly.

### Age-stratified seropositivity

**Fig. 2:**
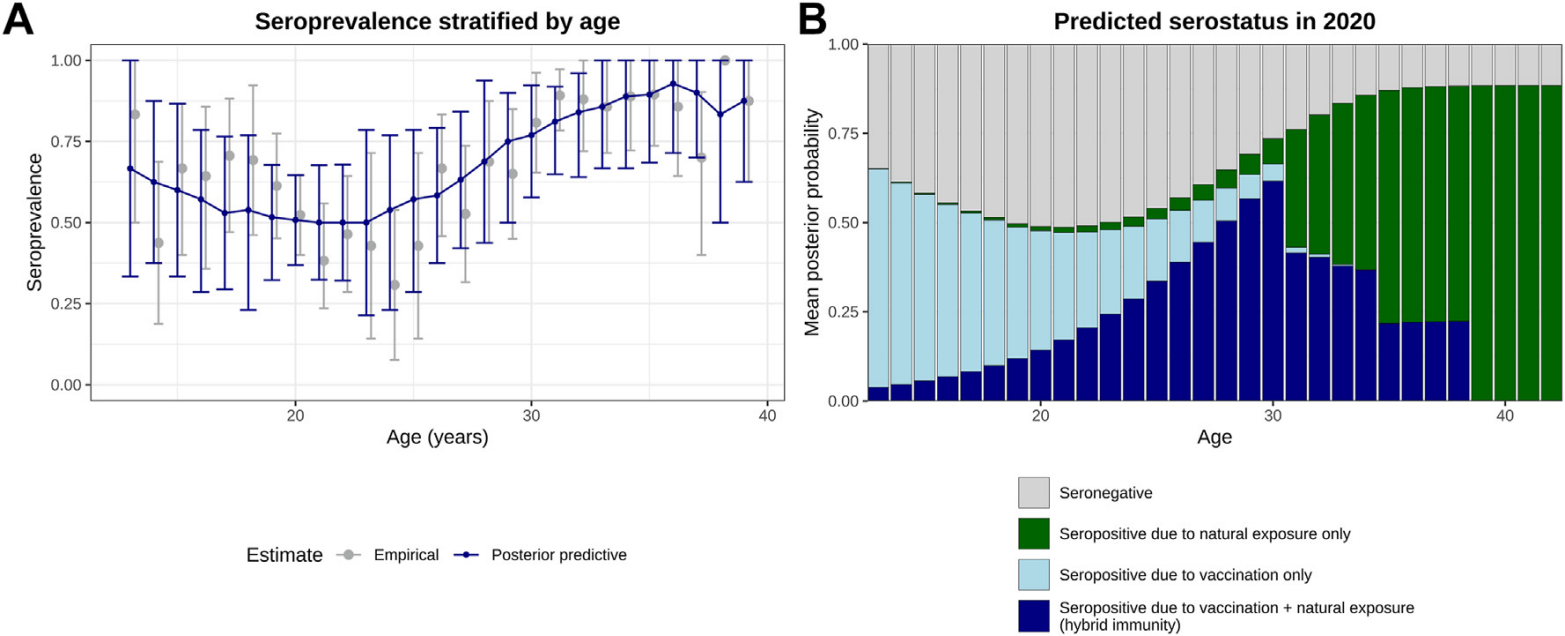
**(A)** Posterior predictive curves for the age-stratified seropositivity derived from a simple model (blue, error bars indicate 95% credible intervals) against empirical values (grey, error bars indicating 95% confidence intervals after bootstrap resampling (8000 iterations) within age groups). **(B)** The posterior mean seropositivity probabilities arising from vaccination, natural exposure, or hybrid immunity in 2020, based on 8000 posterior parameter combinations (sampled uniformly at random without replacement).

Fig. 2A illustrates posterior predictive age-stratified seropositivity curves alongside empirical data. Posterior predictive curves exhibit a biphasic pattern of seropositivity; a decline is observed from ages 13 to 20, followed by an increase from ages 26 to 39. However, the model fails to capture the apparent decline in seropositivity between ages 18 to 24 in the empirical data; as a caveat, we note that the empirical drop in seropositivity between ages 23 and 25 is informed by a limited number of samples (n = 31).

### Serostatus prediction

Fig. 2B depicts our model’s prediction for seropositivity, attributable to vaccination, natural exposure, or hybrid immunity–a combination of both. Seropositivity sub-states are unobservable in practice but can be derived under our model. For the year 2020, the posterior mean serostate probabilities, stratified by age, indicate a progressive decline in the proportion of vaccine-induced seropositivity following childhood immunization. The proportion of seronegative individuals initially increases until the age of 25. Subsequently, a gradual decrease in seronegative group is observed, likely resulting from the acquisition of immunity through natural exposure or the development of hybrid immunity.

### Scenario analysis

**Fig. 3:**
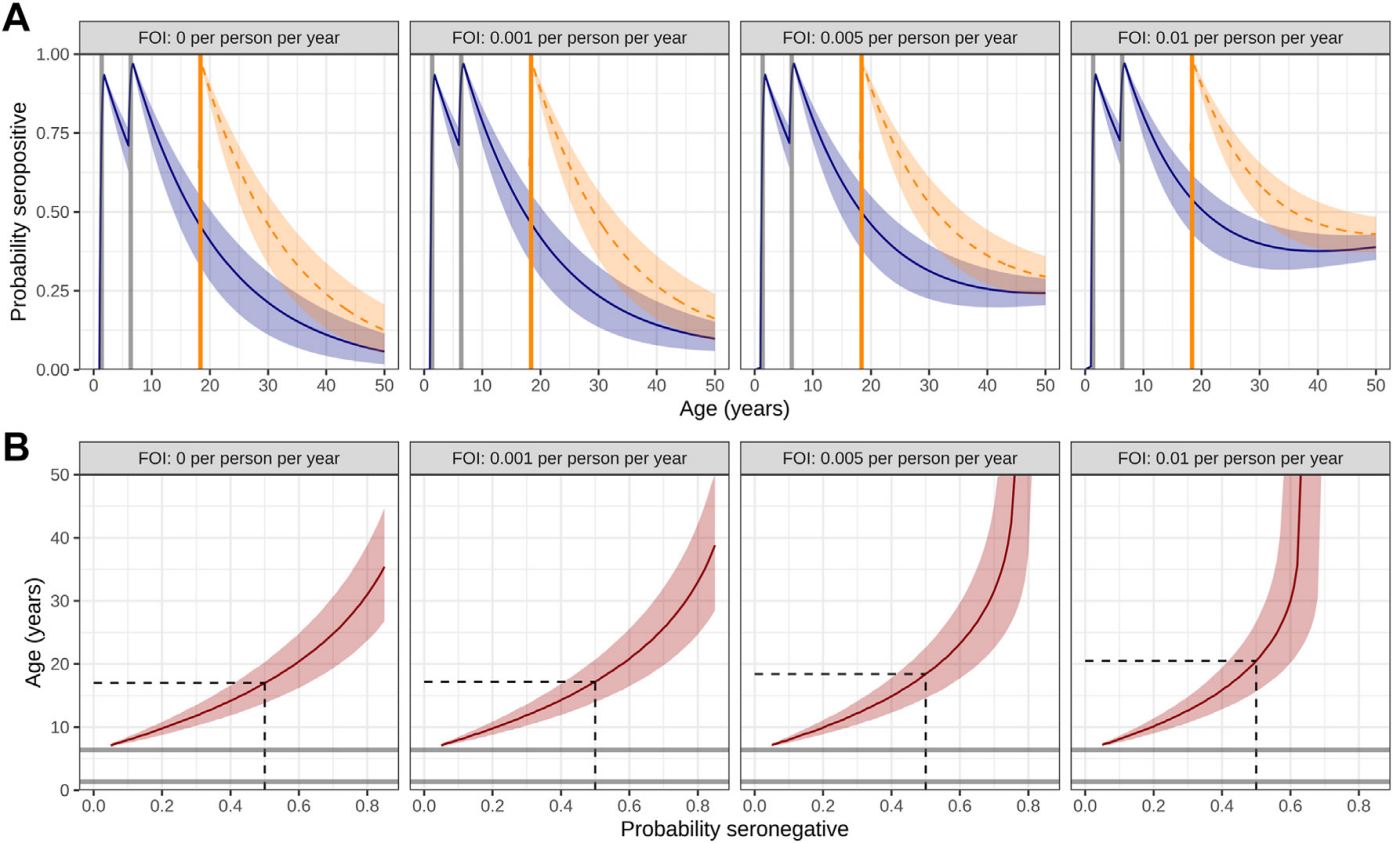
**(A)** Model-derived seropositivity as a function of age given two doses of vaccine are administered at ages one and six, each with a seroconversion probability of 95% (blue); the impact of a third dose at age 18 (likewise with seroconversion probability 95%) is superimposed in orange. **(B)** The age at which different seronegativity thresholds are reached, as a function of the FOI, given the administration of two doses at ages one and six respectively.

We performed a scenario analysis, informed by the estimated time scales of naturally-acquired versus vaccine-induced immunity, for the probability of seropositivity as a function of age. At baseline, we modelled the scenario where two doses of the vaccine are administered–the first at one year and the second at six years of age, each with a seroconversion probability of 0.95–under the simplifying assumption that an individual is subjected to a constant FOI throughout their lifetime. We considered a range of biologically plausible values instead of relying on our estimates for the recent FOI, which are very low due to the assumption of exponential decay and may not be reliably extrapolated. Model-predicted age-stratified seropositivity curves vary at baseline according to annual measles circulation (navy blue, Fig. 3A). Using a maximum seronegativity threshold, we can determine a suitable age range for administering the MCV3 as shown in Fig. 3B. The age at which the probability of seronegativity reaches approximately 50% depends on the FOI (i.e., the rate of natural exposure to measles). For a constant low-level FOI in the range 0–0.01 exposures per person per year on average, 50% seronegativity is typically reached between 18 and 20 years of age; this age range would also apply for time-dependent FOIs fluctuating between 0 and 0.01 exposures per person per year. Here, we have not explicitly modelled high risk groups where outbreaks may occur; instead, we make the argument that MCV3 should be administered to all individuals within a suitable age range. Seropositivity curves given the additional administration of the MCV3 at age 18 (with assumed seroconversion probability 0.95, and an equivalent rate of seroreversion of vaccine-induced immunity to MCV1 and MCV2) are superimposed in orange Fig. 3A.

## Discussion

Since the implementation of a two-dose MCV regimen in 1996, Thailand has maintained high vaccination coverage rates (≥95%) for MCV1.^7^ Despite this, seropositivity rates for measles among adolescents and young adults remain relatively low. This study found that seropositivity rates were generally below 60% in individuals aged 13–20 years and 21–30 years, who should have received two doses of MCV under the Thai EPI. These rates are lower than those observed in a 2014 survey,^18^ yet align with findings from 2020.^25^ The primary reasons for these low rates appear to be insufficient MCV2 coverage, waning of vaccine-derived immunity, or issues with the measles vaccine used in the EPI. To address these issues, it might be beneficial to aim for MCV1 and MCV2 coverage rates as high as ≥95%, similar to those achieved in Singapore, which resulted in a seropositivity rate of 96.8% among children.^26^ Nevertheless, waning immunity in adolescents and young adults is a prominent issue in some highly immunized countries such as Taiwan.^27^ Moreover, in Vietnam, there are significant discrepancies between reported vaccine coverage (90–95%) and estimated seropositivity rates (60–70%),^28^ indicating potential challenges in achieving consistent seropositivity rates across different settings.

This study indicates that young adults (aged 20–26 years), accounting for nearly 40% of all subjects, exhibit the most significant immunity gaps in Thailand with the lowest seropositivity rates, while individuals aged 28 years or older, some of whom have received one dose of MCV, display relatively higher seropositivity. These gaps suggest that ongoing evaluation of immunization programs and booster strategies is important when there are substantial differences in the respective durations of vaccine-induced and naturally acquired immunity. The patterns identified in our study are consistent with various studies,^27^^,^^29^^,^^30^ including those conducted in countries with high vaccine coverage. However, overall seropositive trend to be higher in Western countries compared to Asian countries.^27^ It is possible that individuals over 28 years of age were more likely to contract measles during their childhood after receiving only one dose of MCV. Alternatively, they may have contracted measles during their college or university years–a period characterized by waning vaccine–derived immunity but sustained cellular immunity, indicative of secondary vaccine failure.^31^ In cases where measles infection occurred after prior immunization, the symptoms were likely milder, noncontagious, and elicited a secondary immune response with notably high levels of neutralization.^32^

SIAs targeting adolescents and young adults could potentially address these immunity gaps,^30^^,^^33^ although their efficacy in controlling outbreaks remains uncertain, as effective management requires timely vaccination and prevention strategies in high-risk individuals.^33^ Our results suggests that individuals aged 18–20 years, typically first-year university students, exhibit a seropositivity probability of 50%, making them a prime target for SIAs or a MCV3. The impact of a booster or third dose of Measles-Mumps-Rubella may be beneficial for outbreak control among highly immunized populations.^15^ In countries with limited resources or inadequate surveillance systems, implementing routine MCV3 for all high-risk populations–such as, military recruits, individuals living in crowed areas, or healthcare personnel aged 18–20–could provide significant benefits in controlling outbreaks and eliminating immunity gaps. Additionally, immunity testing prior to vaccination represents a feasible option within clinical settings; however, the expense associated with assessing measles IgG is similar to the cost of administering the MMR vaccine in Thailand (approximately 10 USD). Given the simplicity and likely improved compliance, routine vaccination without prior serological testing could be a more practical approach, potentially enhancing vaccine uptake on a nation scale. For the implementation of vaccination policy, it is crucial to perform cost-effectiveness or budget impact analyses to ascertain whether the routine administration of an MCV3 should be universal or targeted to specific demographic groups. Currently, it is mandatory for all first-year students enrolled in public health-related faculties at Thai universities to receive MCV3 as a component of their routine immunization schedule.

This study has several strengths. Firstly, the model is grounded in contemporary data, incorporating populations with diverse vaccination background within EPI, including scenarios such as a single dose of MCV, two doses with second administered to first grade students, and where MCV is substituted with MMR.^7^ Our outcomes are consistent with findings reported in existing literature.^18^^,^^25^^,^^27^^,^^29^^,^^30^ Secondly, the results provide valuable insights for the public health sector in determining the suitable age range for administering MCV3, thereby informing national vaccination policy. There are some limitations to our study. Firstly, we utilized the ELISA technique for measuring measles IgG in routine services, which exhibits lower sensitivity compared to the gold standard plaque reduction neutralization test (PRNT). Due to fundamental differences between ELISA and PRNT assays, converting ELISA measurements to PRNT titers is not straightforward. Additionally, in this study, seropositivity as determined by ELISA should not be interpreted as indicative of seroprotection. Since our seroprevalence data is derived primarily from a hospital-based sample, its representativeness may be limited, as it predominantly includes healthcare personnel and international students who are subject to antibody testing requirements. Incorporating both community-based and hospital-based seroprevalence data would enhance the overall representativeness of the findings. Lastly, the age range of the study (13–39 years) limits our resolution to characterise early dynamics of antibody waning and boosting. Based on cross-sectional data, we are not equipped to characterise heterogeneity in either seroreversion rates or the initial level of immunity.^34^ Small sample sizes within certain age groups (Supplementary Fig. S4) limit the precision of our age-stratified seropositivity curves, including the magnitude of the apparent decline in seropositivity in the age range 23–25 years. The constructed model does not fully capture empirical data for individuals aged 13–14 or 18–24 years. This discrepancy could potentially be attributed to sporadic outbreaks or fluctuations in transmission (which would not be captured by a model that assumes a pre-defined functional form for the FOI) that could yield erratic age-specific immunity levels in the context of high vaccine coverage.^35^

In conclusion, our study findings highlight a significant measles immunity gap in young adults aged 20–26 years, posing an increased risk of transmission. A MCV3 at the age of 18–20 years potentially closes the gap and enhances measles elimination programmes. Countries can make tailored decisions to effectively address these immunity gaps, either by routine MCV3 implementation for all individuals or by specifically targeting high-risk populations.

## Contributors

**Conceptualization:** Thundon Ngamprasertchai, Wirichada Pan-ngum.

**Data curation:** Somya Mehra, Sajikapon Kludkleeb, Thundon Ngamprasertchai, Wirichada Pan-ngum.

**Formal analysis:** Somya Mehra, Sajikapon Kludkleeb, Thundon Ngamprasertchai, Wirichada Pan-ngum.

**Funding acquisition:** Thundon Ngamprasertchai, Wirichada Pan-ngum.

**Methodology:** Thundon Ngamprasertchai, Saranath Lawpoolsri, Wirichada Pan-ngum.

**Resources:** Chutikarn Chaimayo, Pornsawan Leaungwutiwong.

**Validation and visualization:** Somya Mehra, Sajikapon Kludkleeb, Thundon Ngamprasertchai, Wirichada Pan-ngum, Saranath Lawpoolsri, Kulkunya Chokephaibulkit.

**Writing—original draft preparation:** Somya Mehra, Sajikapon Kludkleeb, Thundon Ngamprasertchai, Wirichada Pan-ngum.

**Writing—review and editing:** All.

Thundon Ngamprasertchai, Wirichada Pan-ngum have directly accessed and verified the underlying data. All authors had full access to the data and the final responsibility to submit for publication.

## Data sharing statement

Data can be made available upon reasonable request to thundon.ngm@mahidol.ac.th.

## Declaration of generative AI and AI-assisted technologies in the writing process

During the preparation of this work, the authors used ChatGPT 4o version to assist with English editing. After using this tool, the authors reviewed and edited the content as needed and take full responsibility for the content of the published article.

## Declaration of interests

The authors declare no conflict of interest. The funders had no role in the design of the study; in the collection, analyses, or interpretation of data; in the writing of the manuscript; or in the decision to publish the results.
