# Supplementary material for: Unveiling immunity gaps and determining a suitable age for a third dose of the measles-containing vaccine: a strategic approach to accelerating measles elimination
Source: Lancet Reg Health Southeast Asia. 2024 Dec 21;32:100523. doi: 10.1016/j.lansea.2024.100523 (PMC11732191; doi:10.1016/j.lansea.2024.100523)
Supplement: Supplementary Material [file mmc1.pdf]

Supporting information for

Unveiling Immunity Gaps and Determining a Suitable Age for a Third Dose of the Measles-Containing Vaccine: A Strategic Approach to Accelerating Measles Elimination

1 Model structure

Here, we construct a serocatalytic dynamic probability model, stratifying seropositivity due to vaccination vs natural exposure for a single individual, born in the year b. At birth, the individual is assumed to be seronegative i.e. maternal immunity is ignored. This is justified because the expected longevity of maternal immunity (in the order of several years) is far smaller than the age range of interest (13 to 39 years).

We account for the administration of $n_{\text{dose}}^{(b)}$ of a measles-containing vaccine. We model dose i of the vaccine to be administered at a constant rate $r_i^{(b)}$ between the ages $A_i^{(b)}$ and $A_i^{(b)} + T$. Seroconversion following vaccination is assumed to be instantaneous. We parameterise the rate $r_i^{(b)}$ based on the probability of seroconversion $c_i^{(b)}$ associated with dose i, computed as the product of vaccine coverage in the year $b + A_i^{(b)}$ and estimated vaccine effectiveness. An elementary calculation then yields

$$r_i^{(b)} = -\frac{\log\left(1 - c_i^{(b)}\right)}{T}.$$

In modelling natural exposure, we allow for time-dependence, but not age-dependence in the force of infection (FOI) $\lambda(t)$. The FOI $\lambda(t)$ parametrises the rate at which an individual is exposed to measles at time t; only non-immune individuals will develop disease upon exposure. This functional form allows us to capture systematic declines in transmission following widespread immunisation programs, but does not account for behavioural variation in exposure amongst age groups (e.g. increased transmission amongst young adults due to crowded conditions in student dorms). For simplicity, we assume a constant FOI $\lambda_{\max}$ prior to the introduction

of the Extended Program of Immunisation (EPI) in 1982. From 1982 onwards, we assume that the FOI decays exponentially at rate α per year, that is,

$$\lambda(t) = \begin{cases} \lambda_{\max} & \text{if } t \leq 1982 \\ \lambda_{\max} e^{-\alpha(t-1982)} & \text{if } t > 1982 \end{cases}. \tag{1}$$

We allow for differing rate of seroreversion for individuals who are seropositive due to naturally exposure w_{natural} vs vaccination only w_{vaccine}. We expect naturally-acquired immunity to have greater longevity than vaccine-induced immunity, that is, $w_{\text{natural}} < w_{\text{vaccine}}$.

1.1 Governing equations

Consider an individual born at time b. At time $t \geq b$, denote by:

- $S^{(b)}(t)$ the probability that the individual is seronegative.
- $P^{(b)}_{\text{vaccine}}(t)$ the probability that the individual is seropositive due to vaccination only;
- $P^{(b)}_{\text{natural}}(t)$ the probability that the individual is seropositive due to natural exposure only;
- $P^{(b)}_{\text{both}}(t)$ the probability that the individual is seropositive due to both natural exposure and vaccination (i.e. hybrid immunity).

Let $H(\cdot)$ denote the Heaviside step function. Then we obtain the governing equations

$$\begin{aligned} \frac{dN^{(b)}}{dt} = &- \underbrace{\lambda(t)N^{(b)}(t)}_{\substack{\text{natural exposure}\\ \text{seroconversion}}} - \underbrace{\sum_{i=1}^{n^{(b)}_{\text{doses}}} r^{(b)}_i H\big(t-b-A^{(b)}_i\big)H\big(b+A^{(b)}_i+T-t\big)N^{(b)}(t)}_{\text{vaccine-induced seroconversion}} \\ &+ \underbrace{w_{\text{vaccine}}P^{(b)}_{\text{vaccine}}(t)}_{\substack{\text{waning of vaccine-}\\ \text{induced immunity}}} + \underbrace{w_{\text{natural}}P^{(b)}_{\text{natural}}(t)}_{\substack{\text{waning of naturally-}\\ \text{acquired immunity}}} + \underbrace{w_{\text{natural}}P^{(b)}_{\text{both}}(t)}_{\substack{\text{waning of hybrid}\\ \text{immunity}}} \end{aligned} \tag{2}$$

$$\frac{dP^{(b)}_{\text{vaccine}}}{dt} = - \underbrace{w_{\text{vaccine}}P^{(b)}_{\text{vaccine}}(t)}_{\substack{\text{waning of vaccine-}\\ \text{induced immunity}}} - \underbrace{\lambda(t)P^{(b)}_{\text{vaccine}}}_{\substack{\text{individual seropositive due}\\ \text{to vaccination only}\\ \text{is naturally exposed}}} + \underbrace{\sum_{i=1}^{n^{(b)}_{\text{doses}}} r^{(b)}_i H\big(t-b-A^{(b)}_i\big)H\big(b+A^{(b)}_i+T-t\big)N^{(b)}(t)}_{\text{vaccine-induced seroconversion}} \tag{3}$$

$$\frac{dP^{(b)}_{\text{natural}}}{dt} = - \underbrace{w_{\text{natural}}P^{(b)}_{\text{natural}}}_{\substack{\text{waning of naturally-}\\ \text{acquired immunity}}} - \underbrace{\sum_{i=1}^{n^{(b)}_{\text{doses}}} r^{(b)}_i H\big(t-b-A^{(b)}_i\big)H\big(b+A^{(b)}_i+T-t\big)P^{(b)}_{\text{natural}}}_{\text{individual seropositive due to natural exposure only is vaccinated}} + \underbrace{\lambda(t)N^{(b)}(t)}_{\substack{\text{natural exposure}\\ \text{seroconversion}}} \tag{4}$$

$$\frac{dP^{(b)}_{\text{both}}}{dt} = \underbrace{-w_{\text{natural}} P^{(b)}_{\text{both}}}_{\substack{\text{waning of hybrid}\\ \text{immunity}}} + \underbrace{\lambda(t) P^{(b)}_{\text{vaccine}}(t)}_{\substack{\text{individual seropositive due}\\ \text{to vaccination only}\\ \text{is naturally exposed}}} + \underbrace{\sum_{i=1}^{n^{(b)}_{\text{doses}}} r^{(b)}_i H\big(t - b - A^{(b)}_i\big) H\big(b + A^{(b)}_i + T - t\big) P^{(b)}_{\text{natural}}(t)}_{\text{individual seropositive due to natural exposure only is vaccinated}} \tag{5}$$

with the initial condition

$$S^{(b)}(b) = 1,\ P^{(b)}_{\text{vaccine}}(b) = P^{(b)}_{\text{natural}}(b) = P^{(b)}_{\text{both}}(b) = 0.$$

Under this initial condition, each individual is modelled to be seronegative at birth with probability one.

Preliminary analyses suggest that the system of ODEs given by Equations (2) to (5) is stiff. To solve this system numerically, we use the backward differentiation formula, as implemented in the R function `deSolve::ode` (with the method set to `"bdf"`) (Soetaert, Petzoldt, and Setzer 2010).

2 Parameter estimation

2.1 Likelihood function

For a cohort of n_{cohort} individuals, indexed $k = 1, \ldots, n_{\text{cohort}}$, our observed data comprise of a series of binarised serostates S_k, with $S_k = 1$ if individual k is seropositive and $S_k = 0$ if individual k is seronegative. We also record the birth year b_k and measurement year t_k for each individual, which uniquely specify the age at vaccination $A_i^{(b_k)}$ and the vaccination rates $r_i^{(b_k)}$ for each dose $i = 1, \ldots, n^{(b_k)}_{\text{dose}}$ of a measles-containing vaccine. We assume that each dose of the vaccine is administered over a period of $T = 0.75$ years, and allow for up to two vaccine doses, at age $A_1^b = 1$ year for $b \geq 1982$ and $A_2^b = 6$ years for $b \geq 1990$. Vaccine regimens are detailed in Appendix 3.1, while vaccine coverage (based on Thai government data) is detailed in Appendix 3.2.

Denote by $N^{(b_k)}(t_k | w_{\text{vaccine}}, w_{\text{natural}}, \lambda_{\max}, \alpha)$ the probability that an individual born in year b_k is seronegative at time t_k for a given set of parameters, and the assumed functional form (1) for the FOI. To recover this quantity, we note that it is sufficient to solve the system of ODEs given by Equations (2) and (3), with the substitution

$$P^{(b)}_{\text{natural}}(t) + P^{(b)}_{\text{both}}(t) = 1 - N^{(b)}(t) - P^{(b)}_{\text{vaccine}}(t).$$

This is because we have assumed the same rate of seroreversion w_{natural} for individuals who are

seropositive due to natural infection only, or both natural infection and vaccination.

We model the serostate S_k of each individual k independently. The likelihood of observing the serostates $\mathbf{S} = (S_1, \ldots, S_{n_{\text{cohort}}})$ takes the form

$$\begin{aligned}&\mathcal{L}(\mathbf{S}|w_{\text{vaccine}}, w_{\text{natural}}, \lambda_{\max}, \alpha, \mathbf{b}, \mathbf{t}) \\ &= \prod_{k=1}^{n_{\text{cohort}}} N^{(b_k)}(t_k|w_{\text{vaccine}}, w_{\text{natural}}, \lambda_{\max}, \alpha)^{1-S_k} \Big(1 - N^{(b_k)}(t_k|w_{\text{vaccine}}, w_{\text{natural}}, \lambda_{\max}, \alpha)\Big)^{S_k}. \quad (6)\end{aligned}$$

By construction, this likelihood function adjusts for variation in sample sizes across age groups.

2.2 Metropolis-Hastings algorithm

We perform parameter estimation in a Bayesian setting using the Metropolis-Hastings algorithm. The likelihood function is given in Equation (6). We take flat improper priors over the range $(0, +\infty)$ for each parameter $\{w_{\text{vaccine}}, w_{\text{natural}}, \alpha\}$, and an informative Gamma prior

$$\lambda_{\max} \sim \Gamma(3, 1/3)$$

(where we have used the shape-scale parametrisation) for identifiability.

We initialise each chain by independently sampling

$$\begin{aligned}w_{\text{vaccine}} &\sim U[1/50, 1/5] \\ w_{\text{natural}} &\sim U[1/100, 1/10] \\ \alpha &\sim U[0.01, 0.3] \\ \lambda_{\max} &\sim \Gamma(3, 1/3).\end{aligned}$$

Given a parameter set $\{w'_{\text{vaccine}}, w'_{\text{natural}}, \alpha'\}$, we sample candidate parameters from the symmetric proposal distributions

$$\begin{aligned}w^*_{\text{vaccine}} &\sim \mathcal{N}^{\mathcal{R}}\big(w'_{\text{vaccine}}, 1/600\big) \\ w^*_{\text{natural}} &\sim \mathcal{N}^{\mathcal{R}}\big(w'_{\text{natural}}, 1/600\big) \\ \alpha^* &\sim \mathcal{N}(\alpha', 1/120) \\ \lambda^*_{\max} &\sim \mathcal{N}^{\mathcal{R}}(\lambda'_{\max}, 0.08)\end{aligned}$$

where $\mathcal{N}(\mu, \sigma)$ denotes the normal distribution with mean ν and standard deviation σ, while $\mathcal{N}^{\mathcal{R}}(\mu, \sigma)$ denotes the corresponding rectified normal distribution (i.e. with all negative values mapped to zero).

We run the algorithm for 4 chains, each spanning 80000 iterations. The first 10000 iterations of each chain are excluded as the burn-in period.

Parameter	Interpretation	Posterior [95% CrI]
$1/w_{\text{vaccine}}$	Average duration of vaccine-induced immunity	15.3 [10.8, 20.2] years
$1/w_{\text{natural}}$	Average duration of naturally-acquired immunity	208 [119, 418] years
λ_{max}	Force of infection before immunisation program implemented (before 1982)	1.8 [0.75, 3.7] year^{-1}
α	Rate of decay of force of infection (exponential) after immunisation program implemented (1982 onwards)	0.21 [0.15, 0.28] year^{-1}

Table 1: Summary of parameter estimates, including posterior medians and 95% credible intervals (CrI)

2.3 Assessing convergence

To assess convergence, we visually inspect trace plots (Figure 1). We additionally compute the Gelman-Rubin diagnostic R for each estimated parameter, by evaluating Equation (1) of Brooks and Gelman (1998) after excluding the burn-in period. Marginal posterior densities, stratified by chain, are shown in Figure 2.

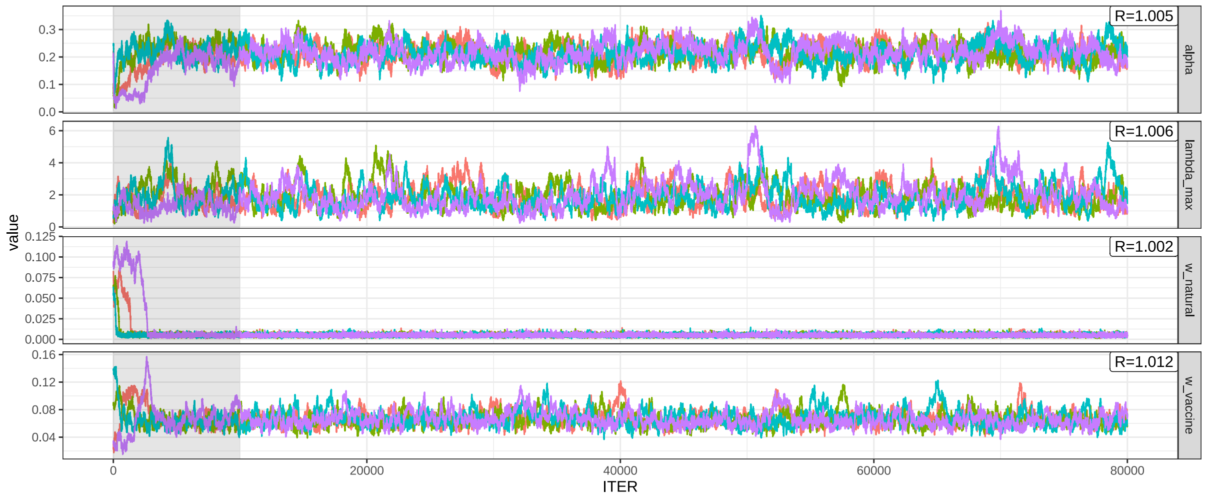

Figure 1: Trace plots for the Metropolis-Hastings algorithm, with the burn-in period shown in grey.

2.4 Pairwise posteriors

Marginal posterior estimates are summarised in Table 1. An examination of pairwise posteriors reveals strong correlation structure, particularly between the FOI λ_{max} before 1982 and the (exponential) rate of decay α of the FOI thereafter (Figure 3). The expected duration of naturally-induced immunity $1/w_{\text{natural}}$ is estimated to be lifelong. Under this setting, the probability of naturally-acquired immunity is a direct function of cumulative exposure; the model thus

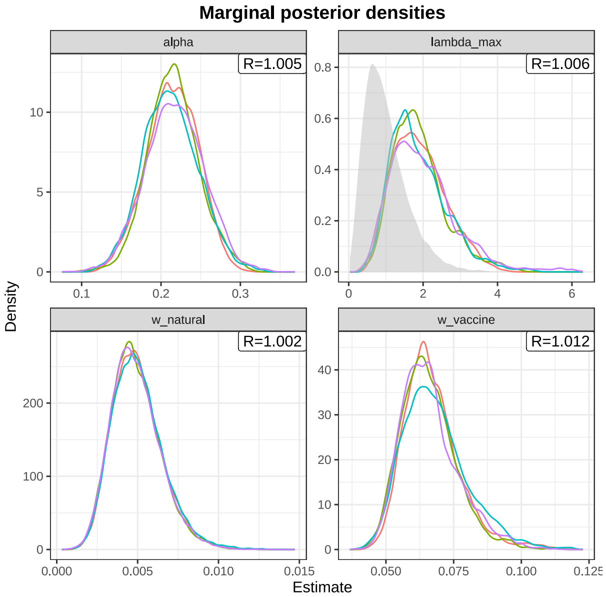

Figure 2: Marginal posteriors for each estimated parameter. Densities are stratified by chain. The prior distribution for $\lambda_{\max}$ is shown in grey; for all other parameters, we take an improper flat prior on the positive real line.

yields similar age-stratified seropositivity profiles under a low initial FOI that decays slowly; or a high initial FOI that decays rapidly.

2.5 Posterior predictive checks

To perform posterior predictive checks and quantify the relative contributions of natural exposure vs vaccination in driving seropositivity, we sample 8000 posterior parameter combinations (uniformly at random, without replacement), and solve the complete system of ODEs given by (2) to (5) for each parameter set. To yield posterior predictive age-stratified posterior curves, we then simulate

$$\text{Seroprevalence}(a) = \frac{1}{n(a)} \sum_{i=1}^{n(a)} X_i(a)$$

where

$$n(a) = \sum_{k=1}^{n_{\text{cohort}}} \mathbb{1}\{t_k - b_k = a\}$$

$$X_i(a) \overset{\text{i.i.d.}}{\sim} \text{Bernoulli}\left(\sum_{k=1}^{n_{\text{cohort}}} N^{(b)(b+a)} \cdot \frac{\mathbb{1}\{t_k - b_k = a, b_k = b\}}{n(a)} \right).$$

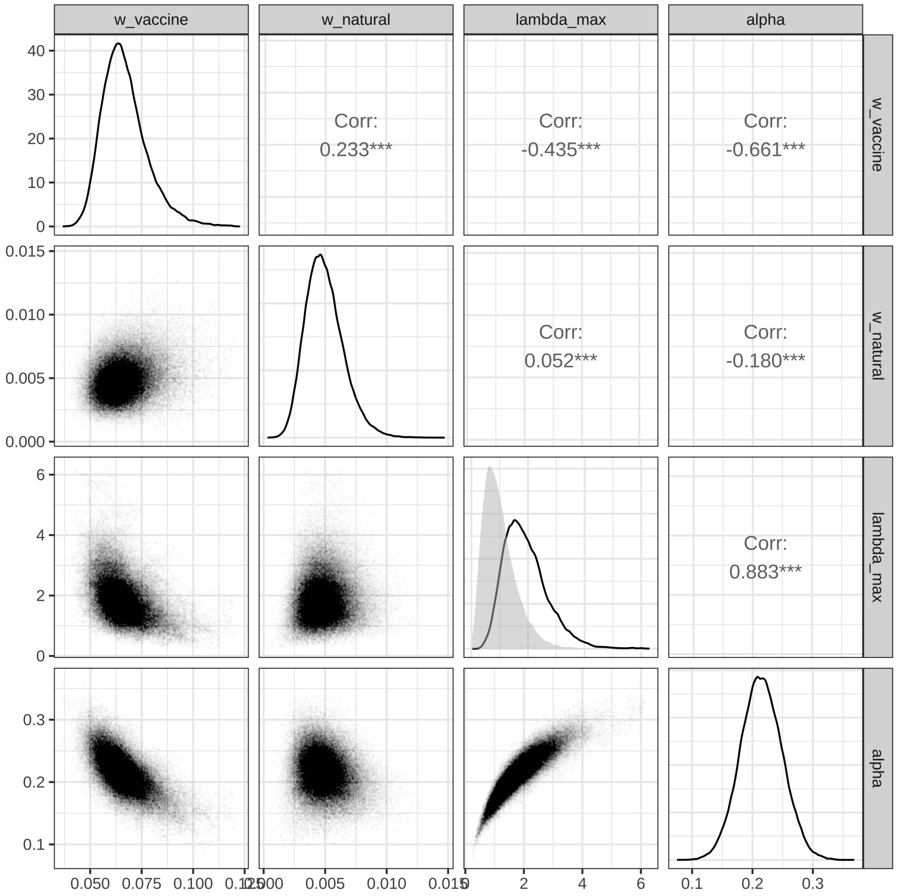

Figure 3: Pairwise summary of posterior estimates. Marginal posteriors are shown along the diagonal; the informative prior distribution for $\lambda_{\max}$ is shaded in grey. Plots have been generated using the R function `GGally::ggpairs`.

This construction ensures that the probability of seropositivity for each age group a is weighted by birth and measurement year in the same way as empirical data when bootstrapping resampling. It also reflects uncertainty due to sample size variation across age groups.

References

Brooks, Stephen P and Andrew Gelman (1998). "General methods for monitoring convergence of iterative simulations". In: *Journal of computational and graphical statistics* 7.4, pp. 434–455.

Soetaert, Karline, Thomas Petzoldt, and R Woodrow Setzer (2010). "Solving differential equations in R: package deSolve". In: *Journal of statistical software* 33, pp. 1–25.

3 Supplementary data

3.1 Vaccine regimens

Table 2: Vaccine regimens under the Thai Extended Program of Immunisation (EPI). Vaccine effectiveness is assumed to be 0.9 for the monovalent measles-containing vaccine (M) and 0.95 for the trivalent measles-containing vaccine (MMR).

Birth year	Dose 1 type	Dose 1 year	Dose 2 type	Dose 2 year
1978	–	–	–	–
1979	–	–	–	–
1980	–	–	–	–
1981	–	–	–	–
1982	M	1983	–	–
1983	M	1984	–	–
1984	M	1985	–	–
1985	M	1986	–	–
1986	M	1987	–	–
1987	M	1988	–	–
1988	M	1989	–	–
1989	M	1990	–	–
1990	M	1991	M	1996
1991	M	1992	MMR	1997
1992	M	1993	MMR	1998
1993	M	1994	MMR	1999
1994	M	1995	MMR	2000
1995	M	1996	MMR	2001
1996	M	1997	MMR	2002
1997	M	1998	MMR	2003
1998	M	1999	MMR	2004
1999	M	2000	MMR	2005
2000	M	2001	MMR	2006
2001	M	2002	MMR	2007
2002	M	2003	MMR	2008
2003	M	2004	MMR	2009

2004	M	2005	MMR	2010
2005	M	2006	MMR	2011
2006	M	2007	MMR	2012
2007	M	2008	MMR	2013
2008	M	2009	MMR	2014

3.2 Vaccine coverage

Table 3: Coverage for measles-containing vaccines by year, based on Thai government data

Year	Reported Coverage	Imputed coverage
1983	–	0.30
1984	–	0.30
1985	–	0.30
1986	0.30	0.30
1987	0.52	0.52
1988	0.56	0.56
1989	–	0.63
1990	0.70	0.70
1991	0.75	0.75
1992	0.74	0.74
1993	0.69	0.69
1994	0.65	0.65
1995	0.85	0.85
1996	0.92	0.92
1997	0.92	0.92
1998	–	0.94
1999	0.96	0.96
2000	0.94	0.94
2001	0.94	0.94
2002	0.94	0.94
2003	0.96	0.96
2004	0.96	0.96
2005	0.96	0.96

2006	0.96	0.96
2007	0.96	0.96
2008	0.98	0.98
2009	0.98	0.98
2010	0.98	0.98
2011	0.98	0.98
2012	0.98	0.98
2013	0.99	0.99
2014	0.99	0.99
2015	0.99	0.99
2016	0.99	0.99

3.3 Serostates stratified by age

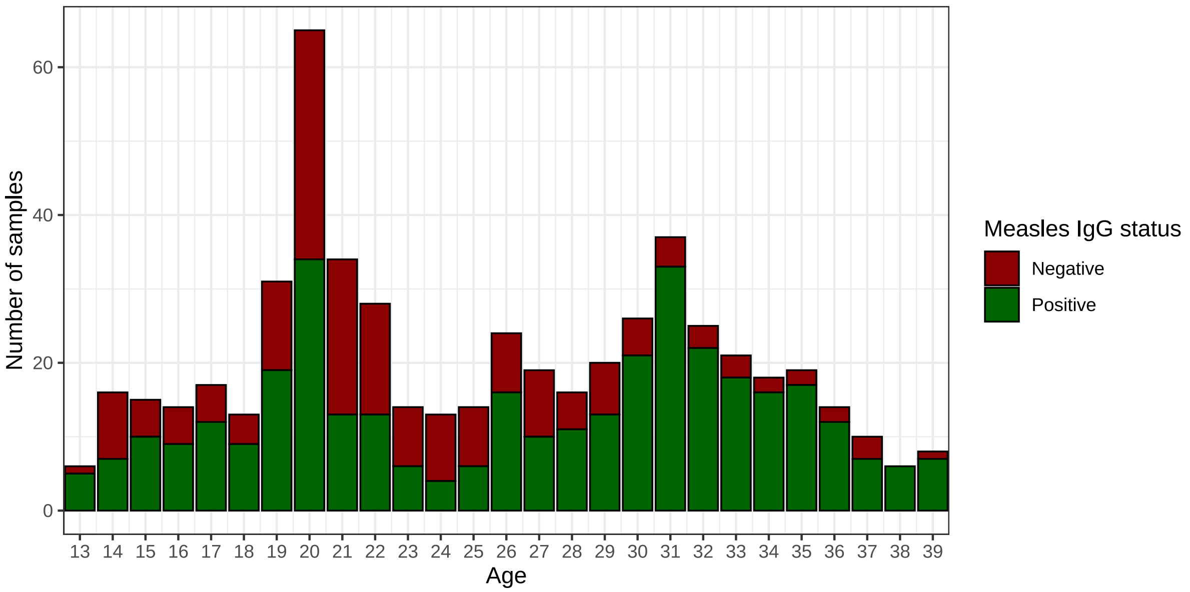

Figure 4: Stacked barplot of serostates and sample counts stratified by age at the time the blood sample was collected; since data were collected between 2018 and 2021, there is a mixture of birth years within each age group.
